# The PACAP-derived peptide MPAPO facilitates corneal wound healing by promoting corneal epithelial cell proliferation and trigeminal ganglion cell axon regeneration

**DOI:** 10.7150/ijbs.35630

**Published:** 2019-10-15

**Authors:** Zixian Wang, Wailan Shan, Huixian Li, Jia Feng, Shiyin Lu, Biqian Ou, Min Ma, Yi Ma

**Affiliations:** 1 Institute of Biomedicine, Department of Cellular Biology, Jinan University; 2 National engineering research center of genetic Medicine, Key laboratory of Bioengineering Medicine of Guangdong Province, Jinan University; 3 College of traditional Chinese Medicine, Jinan University

**Keywords:** PAC1 receptor, corneal wound healing, pituitary adenylate cyclase-activating polypeptide (PACAP), corneal epithelial cells, Wnt/β-catenin signaling pathway

## Abstract

It is well known that the cornea plays an important role in providing protection to the eye, but it is fragile and vulnerable. To clarify the biological effects and molecular mechanisms of the pituitary adenylate cyclase activating polypeptide (PACAP)-derived peptide MPAPO (named MPAPO) to promote corneal wound healing, we applied a mechanical method to establish a corneal injury model and analyzed the repair effects of MPAPO on corneal injury. MPAPO significantly promoted corneal wound repair in C57BL/6 mice. In addition, we established injury models of epithelial cells and trigeminal ganglion cells with H_2_O_2_. The results show that when the concentration of MPAPO is 1 μM, it can significantly promote the repair of injured corneal epithelial cells and the regeneration of trigeminal ganglion cell axons. MPAPO repairs epithelial cells through the promotion of GSK3β phosphorylation by binding to PAC1 and activating AKT. β-catenin escapes the phosphorylation of GSK3β and enters the nucleus to promote the expression of cyclin D1, accelerate cell cycle progression and promote cell proliferation. MPAPO promotes axonal regeneration by binding to the PAC1 receptor and activating adenylate cyclase activity, followed by the cAMP activation of protein kinase A activity and the promotion of CREB phosphorylation. Phosphorylated CREB promotes Bcl_2_ expression and axonal regeneration. In conclusion, our data support the role of MPAPO to facilitate corneal wound healing by promoting corneal epithelial cell proliferation and trigeminal ganglion cell axon regeneration.

## Introduction

The cornea, which is the transparent front part of the eye, plays a role in maintaining the shape of the eyeball and protecting its internal structure. It is convex and covers the iris, pupil and anterior chamber. The cornea can be divided from the outside to inside into the epithelium, lamina elastica anterior (Bowman membrane), stroma, lamina elastica posterior (Descemet membrane) and endothelium 1. Because the cornea is exposed to the external environment, it is vulnerable to injury and infection. Corneal disease is the fourth most common cause of blindness after cataracts 2-4, glaucoma 5 and age-related macular degeneration 6, 7. Corneal wound healing is a complex physiological process involving the repair of epithelial layers, cell migration associated with wound repair, and proliferation of cells associated with tissue regeneration 8.

Pituitary adenylate cyclase-activating polypeptide (PACAP) belongs to a peptide hormone superfamily that includes vasoactive intestinal peptide (VIP), glucagon, and glucagon-like peptides 9, which exists in the form of PACAP38 and PACAP27 *in vivo*. PACAP27 and PACAP38 have the same conformation and similar physiological functions except for the same N-terminal sequence 10. There are two types of PACAP receptors currently found. The type I PACAP receptor (PAC1) has a high affinity for PACAP and a low affinity for VIP. There are two types of type II PACAP receptors, VPAC1 and VPAC2, which have very high affinity for both PACAP and VIP 11, 12. PACAP and its receptors are widely distributed in the central nervous system and other tissues, and play an important role in the development of the body, the development of the nervous system and the repair of nerve endings 13. PACAP and the PACAP receptors are localized in the ocular structures of the retina, uvea, trigeminal ganglion cells, iris sphincters, ciliary bodies and cornea 14-17.

Studies have shown that PACAP can stimulate lacrimal secretion, protect the retina from diabetes damage, and promote the repair of corneal wounds 18-21. However, PACAP has poor stability *in vivo*, and its half-life is less than 10 min. The reason that PACAP is rapidly degraded *in vivo* is the presence of the proteolytic enzyme dipeptidyl peptidase IV, which rapidly hydrolyzes PACAP into short peptides and amino acids, thus limiting the use of PACAP 22, 23. According to the short half-life of PACAP *in vivo* and the characteristics of its receptor binding site, our group previously designed the PACAP27-derived mutant peptide and named it MPAPO 24. MPAPO has the following advantages: first, it has better stability, more stable activity and a longer half-life than PACAP27, making it more effective for the long-term exertion of its biological activity; second, the structure of the type II PACAP binding site was destroyed in an attempt to retain the structure of the type I PACAP binding site as much as possible, so that the binding and specific agonistic effects of MPAPO to the PAC1 receptor are significantly enhanced. The combination of PACAP and PAC1 activates multiple signaling pathways and exerts different biological functions 25. The classical signaling mechanisms currently recognized include the following: the cAMP-dependent PKA pathway, the cAMP-dependent Epac pathway, the PLC and PKC signaling pathways, and the via PLC, IP3 , Ca^2+^ signaling pathways 26, 27.

Wnt signaling is involved in all aspects of embryonic development, maintains tissue homeostasis and controls tissue self-renewal 28. The Wnt signaling pathway is activated in three distinct ways: the classic Wnt/β-catenin signaling pathway, the planar cell polarity (PCP) signaling pathway and the Wnt/Ca2+ signaling pathway 29. Previous studies have reported that activation of Wnt/β-catenin signaling by simvastatin can inhibit neuronal apoptosis and promote motor function recovery 30. The PAC1 receptor could protect cells from apoptosis or promote cell proliferation via the Wnt/β-catenin pathway 31. In neurons, PACAP activates related signaling pathways through PAC1 receptors, promotes neuronal survival, inhibits neuronal cell apoptosis, and protects neurons with neurotrophic and protective effects. PAC1 could promote neuronal differentiation by activating cAMP and PKA signaling pathways 25. Studies have shown that CREB is a core participant in the promotion of spinal axon regeneration *in vivo* 32. Bcl_2_ regulates the intrinsic growth response of CNS neurons through intracellular Ca2+ signaling and CREB- and Erk-mediated transcriptional programs 33.

Injury of the cornea is inevitably accompanied by injury to the corneal epithelium and the corneal nerve. Research on corneal wound healing at home and abroad is often only one-sided study. In view of the deficiencies of these studies, combined with the strength of the neuropeptides in this group, MPAPO was explored at the animal level to promote corneal wound repair, including corneal epithelial repair and axonal regeneration of the corneal nerve. At the cellular level, the effects of MPAPO on the repair and the promotion of axon regeneration were researched, and the possible mechanisms were explored.

## Materials and Methods

### Animals and treatment

All experiments on animals were in accordance with the use of laboratory animals and the ARVO Statement for the Use of Animals in Ophthalmic and Vision Research. The housing and related procedures were approved by the Laboratory Animal Ethics Committee of Jinan University, China. All animal experiments and euthanasia met animal ethical requirements, and every effort was made to minimize pain and suffering. C57BL/6 mice (SPF grade, male, 8 weeks old, weighing approximately 30 g) were purchased from Guangdong Medical Laboratory Animal Center, examined, and it was confirmed that the mice had no disease in their eyes. The mice used for the experiment were kept in a sterile environment at a temperature of 21±0.5°C, a humidity of 50±10%, and a natural diet.

Sixty C57BL/6 mice were randomly divided into four groups. After establishing the corneal injury model as previously described 34, mice were treated with 0.9% NaCl, 10 μM recombinant bovine (rb)-bFGF, 10 μM PACAP27 or 10 μM MPAPO every 4 h. For each administration group, eye drops (50 μL) were applied topically to both eyes of each mouse until they were euthanized. At 0 h, 12 h, 24 h, 36 h and 48 h after surgical injury, the injured corneas were stained with 1% sodium fluorescein for 3 s, rinsed with sterile saline solution, and quickly photographed with a digital camera (Eastman Kodak, Rochester, NY, USA), recording and observing the repair effect of each group on the cornea. The repair effect of each group on the cornea after administration was analyzed by image analysis software Image-Pro Plus ver. 4.0 (Planetron, Tokyo, Japan), and the repair rate after corneal injury was calculated. (Corneal wound repair rate = wound area after drug repair (S) / wound area after surgical injury (S0) ×100%).

### Hematoxylin and eosin (HE) staining

HE staining was used to visualize corneal wound repair. The eyeball was fixed in tissue fixative for 24 h at room temperature, and then the eyeball was removed, dehydrated, embedded in paraffin and cut into 4-μm-thick sections. The nucleus was stained with hematoxylin and the cytoplasm was stained with eosin, and the samples were then dehydrated, sealed, observed under an optical microscope, and the image was collected and analyzed.

### Cell culture

Mouse corneal epithelial cells and corneal epithelial cell complete medium were purchased from Jiangsu Jiangyin Qishi Biotechnology Co., Ltd. The culture conditions for the corneal epithelial cells were in an incubator supplied with 95% air and 5% CO_2_ at 37°C. The medium was replaced every 2 days, and cells were subcultured by treatment with 0.05% trypsin-EDTA (Invitrogen, Carlsbad, CA, USA). Cells were cryopreserved in a frozen solution containing 500 μL of medium, 400 μL of fetal bovine serum (Thermo Fisher Scientific, Waltham, MA, USA), and 100 μL of DMSO (Sigma-Aldrich, St. Louis, MO, USA). The mouse primary trigeminal ganglion cells were kindly provided by the Eye Hospital of Sun Yat-Sen University, China and were maintained in B27 Dulbecco's modified Eagle's medium (Gibco, Carlsbad, CA, USA) supplemented with 10% fetal bovine serum and 2% serum-free culture factor B27 (Thermo Fisher Scientific, Waltham, MA, USA). The cells were incubated at 37°C in a humidified incubator containing 5% CO_2_ and the medium was replaced every 3 days.

### Cell viability assay

The cell viability in each experimental group was determined by MTT assay (Sigma-Aldrich, St. Louis, MO, USA). Briefly, corneal epithelial cells (10^5^ cells/ml) were seeded in 96-well plates and incubated to reach approximately 70% confluence. The cells were treated with different concentrations (0, 1, 10, 100, 1000 or 10000 nM) MPAPO for 48 h. Then water-soluble tetrazolium salt reagent solution (10 μL) was added to each well followed by immediate incubation for 4 h at 37°C. After discarding the medium and MTT, 150 μL of DMSO was added to each well followed by incubation for another 10 min. Then, the absorbance was measured at a wavelength of 490 nm. The oxidative injury corneal epithelial cell model was established with H_2_O_2_. Cells were treated with different concentrations of H_2_O_2_ (6.25, 25, 100, 400, 1600 or 6400 μM) for 4 h, and the treatment without H_2_O_2_ was used as a control. The condition of cell viability of approximately 50% was selected for the establishment of a corneal epithelial cell model of H_2_O_2_ injury. In the experiments to evaluate the ability of MPAPO to promote cell wound repair, there were five experimental groups: normal control, PBS, PACAP27, MPAPO and MPAPO+MAX.D.4 (Sigma Aldrich, Shanghai, China) groups. The cells were preincubated with MAX.D.4 (a PAC1 receptor antagonist) for 30 min at 37°C 35, and then MPAPO was added. The proliferation rate of the cells in each experimental group was determined using the MTT assay.

### Cell cycle analysis *in vitro*

The effects of MPAPO on the cycle progression of H_2_O_2_-injured corneal epithelial cells was evaluated by PI single-staining-flow. Logarithmic growth phase cells (10^5^ cells/ml) were seeded in a 6-well plate and incubated to reach approximately 70% confluence at 37°C, in 5% CO_2_. The oxidatively injured corneal epithelial cells were treated with 1 μM PBS, PACAP27, MPAPO or MPAPO+MAX.D.4 for 4 h. The cells were digested with 0.05% trypsin-EDTA for 2 min at 37°C and harvested by centrifugation. The harvested cells were fixed overnight at 4°C with precooled 70% ethanol. Then, the fixed cells were stained with 20 mg/ml PI stain (Beyotime, Shanghai, China) for 30 min in the dark at 4°C. Finally, the cell cycle was analyzed using a flow cytometer (BD Biosciences), and the red fluorescence at an excitation wavelength of 488 nm was recorded. A DNA histogram of the cell population in the G0/G1, S, and G2/M phases was generated by flow cytometry with CellQuest.

### RNA extraction, reverse transcription, and quantitative RT-PCR

Total RNA was extracted from the cells using TRIzol reagent according to the manufacturer's instructions (Takara, Dalian, China). The total RNA concentration was determined by measuring the optical density at 260 nm with BioPhotometer Plus (Eppendorf, Hamburg, Germany). The genomic DNA knock-out reaction was used to remove the DNA and purify the total RNA following the manufacturer's instructions. Then, 1 μg of RNA was converted into first-strand cDNA using oligo (dT) primer and reverse transcription kit (Takara). Quantitative PCR was performed in a 20 μL reaction mixture containing 1 μL of cDNA, forward primer, reverse primer, and 10 μL of SYBR Green PCR Master Mix (Takara). Each sample was analyzed in triplicate. The three-step PCR procedure was used. After the reaction was complete, the melting curve and the amplification curve are confirmed to be consistent with the requirements. The amplified Ct value was recorded, and the magnitude of 2^-ΔΔCt^ was calculated to speculate the fold difference of the gene expression between the different groups.

### Protein extraction and Western blotting

To explore the expression levels of the proteins associated with various signaling pathways, Western blotting was performed as follows. The harvested cells were lysed with RIPA lysis buffer (Beyotime, Shanghai, China) containing 1% protease inhibitor, 1% phosphatase inhibitor and 1% phenylmethanesulfonyl fluoride [PMSF]. The collected supernatant after centrifugation was the total cellular protein, and then the total protein concentration was determined by the Bicinchoninic Protein Assay Kit (Thermo Fisher Scientific, Waltham, MA, USA) according to the manufacturer's instructions. Equal amounts of protein (~20 mg) were separated by 12% SDS-PAGE and transferred onto polyvinylidene difluoride membranes. The PVDF membrane transfected with protein was placed in a 5% BSA blocking solution and incubated for 1-2 h at room temperature. After blocking, the PVDF membrane was incubated with primary antibody diluted with 2% BSA at 4°C overnight. The primary antibody used had a dilution concentration of 1:1000 and was as follows: β-actin (CST), Akt (CST), p-Akt (CST), GSK3β (CST), p-GSK3β (CST), β-catenin (CST), cyclin D1 (CST), Lamin B1 (CST), Rb (CST), p-Rb (CST), E2F1 (Abcam), CREB (Abcam), p-CREB (Abcam), and Bcl_2_ (CST). The next day, the membrane was washed 5 times with TBS containing 0.1% Tween-20 for 8 minutes each time. Then, the membrane was incubated for an additional hour at room temperature with the appropriate horseradish peroxide-conjugated secondary antibody. After washing with TBST, protein bands were visualized using West Pico Chemiluminescent Substrate (Thermo Fisher Scientific, Waltham, MA, USA). The gray-scale analysis of the Western blotting results was performed using ImageJ software (National Institutes of Health, Bethesda, MD, USA).

### Immunofluorescence

Immunofluorescence staining was used to observe the axonal regeneration of the corneal nerve, MPAPO and optic nerve specific binding, trigeminal ganglion cell wound repair and axonal regeneration, and the entry of β-catenin into the nucleus. To observe axonal regeneration of the corneal nerve, five groups were set up: NaCl, normal control (no injury and no administration), PACAP27, MPAPO and rb-bFGF groups, and the eyeballs were taken out after 48 h injury. The cornea was dissected, fixed in 4% paraformaldehyde for 30 min, and then incubated in 0.25% Triton X-100 for 30 min at room temperature. The cornea was blocked in 5% BSA (dissolved in PAST) for 1 h at room temperature, and then incubate separately with primary antibody (III-β-tublin (Abcam) dilution ratio 1: 50) and secondary antibody (AF594 (CST) dilution ratio 1: 200). The dye was added for 10 min of incubation followed by a 1 h incubation with anti-fluorescence quenching tablets. Finally, the fluorescence results were examined under a laser confocal microscope. To study the specific binding of MPAPO to the optic nerve, the sliced sample was prepared first and then subjected to antigen retrieval. After blocking the sample with 5% BSA, the sample was incubated with the primary (NF200 (Abcam), dilution ratio 1:200) and secondary antibodies (Cy5 (CST), dilution ratio 1:200) separately. Finally, the results were observed under a fluorescence microscope.

To observe trigeminal ganglion cell injury repair and axonal regeneration, mouse trigeminal ganglion cells were seeded in a culture dish, and the cells were oxidatively damaged with H_2_O_2_ when the cells reached approximately 40-50% confluence. After 6 h, the cells were fixed in 4% paraformaldehyde, blocked with 5% BSA, and incubated with the III-β-tublin primary antibody and the secondary antibody separately. DAPI staining was performed to identify the nucleus. Immunofluorescence was used to verify the entry of β-catenin into the nucleus. The cells were seeded in a confocal dish, and when the cell density reached 70-80%, hydrogen peroxide was used for 4 h to damage the cell. Finally, immunofluorescence staining and observation were performed.

### Statistical analysis

Quantitative results are expressed as the mean ± SD of at least 3 independent experiments. All data in this study were statistically analyzed using SPSS19.0 and GraphPad Prism 6 software. Statistical differences between two groups were determined by Student's t test. One-way ANOVA were used for comparisons among different groups. *A P* value <0.05 was considered statistically significant.

## Results

### MPAPO promotes the repair of corneal wounds in C57BL/6 mice

**Figure 1:**
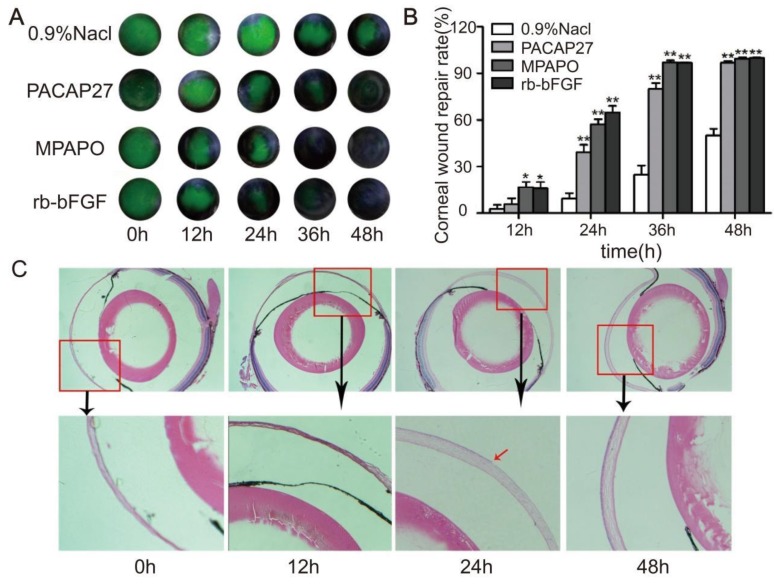
MPAPO promotes the repair of corneal epithelial in C57BL/6 mice. **(A)** MPAPO efficiently promotes corneal epithelial wound closure. Sodium fluorescein staining was used to observe corneal epithelial wound repair in different treatment groups.** (B)** The rates of injured corneal repair in 0.9% NaCl, PACAP27, rb-bFGF and MPAPO-treated mice. **(C)** HE staining was used to observe the effect of MPAPO on the repair of corneal wounds at different times (0 h, 12 h, 24 h, 48 h). *P<0.05, **P<0.01 compared to the 0.9% NaCl group. n=3.

A mouse corneal injury model was established, and the green circular area in the middle of the eye is the cornea after injury (Supplementary Figure 1). C57BL/6 mice with central corneal wounds were treated with 0.9% NaCl, 10 μM MPAP27, MPAPO or rb-bFGF. The results showed that the green corneal wound area in each experimental group decreased at different rates over time. At 36 h after administration, the MPAPO and rb-bFGF treatment groups showed almost complete wound repair; however, the repair rates were only 24.73% and 79.94% in the NaCl- and PACAP27-treated groups, respectively. The NaCl treatment group had the slowest repair rate and was only halfway repaired when the other groups completed their repair at 48 h. (Fig. 1A, 1B).

To further observe the repair of the corneal epithelial wounds, we observed the repair at the microscopic level by HE staining. The results show that the length of the corneal wound arc becomes shorter with increasing time after administration. The changes in corneal epithelial structure were not obvious within 12 h; however, the corneal epithelial layer had grown on approximately half of the corneal stroma after 24 h, and the corneal structure was substantially changed. The cornea was much thicker after 24 h than after 12 h, and the corneal stroma had also been constructed. The corneal epithelium was completely repaired within 48 h after administration, and the intact structure of the entire cornea was clearly observable. The dense corneal epithelial cell layer, the intermediate sponge-like matrix layer and the dense endothelial layer were arranged from the inside to the outside (Fig.1C).

### MPAPO promotes axonal regeneration of the corneal nerve after injury

**Figure 2:**
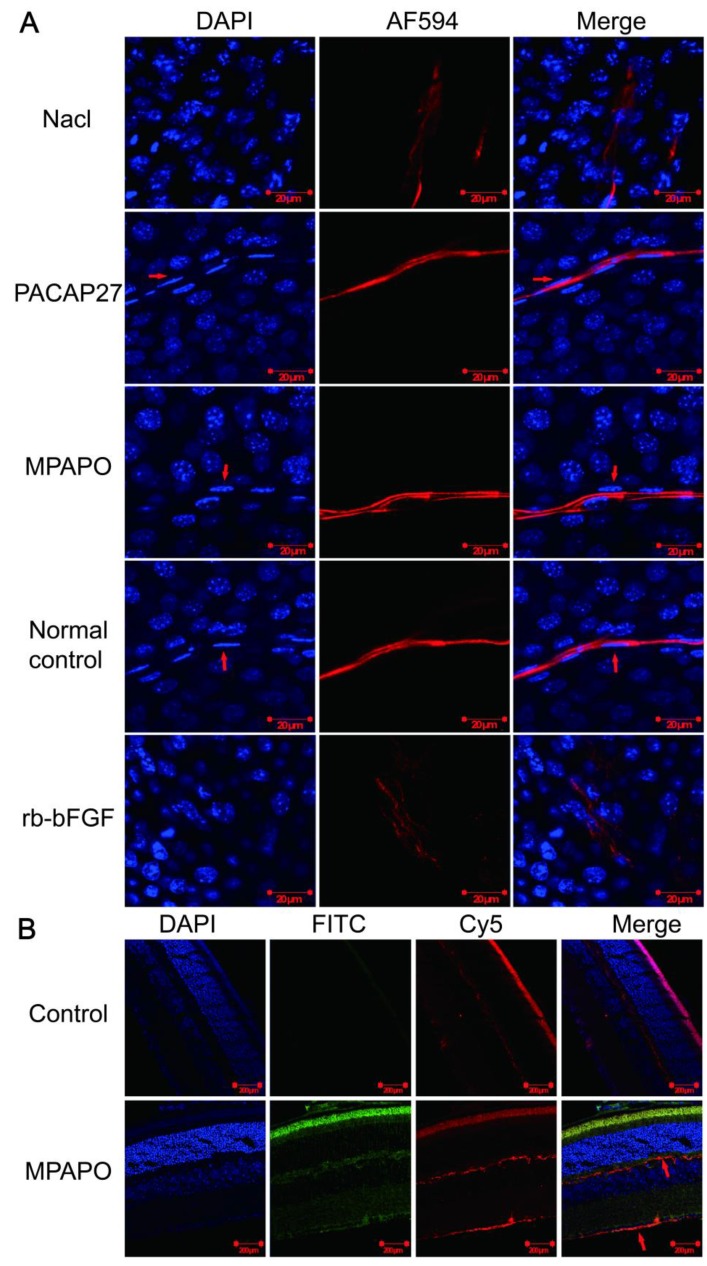
MPAPO promotes axonal regeneration of the corneal nerve after injury in C57BL/6 mice. **(A)** MPAPO promotes axonal regeneration of the corneal nerve as shown by immunofluorescence staining. DAPI is the blue in the nucleus, and AF594 is an anti-mouse red fluorescent secondary antibody used to stain trigeminal nerve fibers (the primary antibody used is the trigeminal tag antibody III-β-tublin). Merge is the result of the colocalization of the two kinds of fluorescence. **(B)** Cross-cut immunofluorescence staining of the eye tissue revealed that MPAPO can bind to optic nerve cells and other retinal cells. DAPI is a nuclear dye with blue fluorescence. FITC is connected to MPAPO with green fluorescence. Cy5 is an anti-rabbit red fluorescent secondary antibody, and the primary antibody is the commonly used neuro-tag antibody NF 200. Merge is the result of the three fluorescent colocalizations.

Immunofluorescence staining verified whether MPAPO can promote axonal regeneration of the corneal nerve after injury at the tissue and individual levels. The results showed that the PACAP27 and MPAPO groups had similar nerve fibers to those of the normal control group (no injury and no administration). The trigeminal nerve fibers are strip-shaped and have a clear structure, and the Schwann cells on the nerve fibers (the position indicated by the red arrow) can be clearly observed on both sides of the nerve fiber cells. The results of the rb-bFGF group were consistent with the 0.9% NaCl group. The trigeminal nerve fibers are diffuse, and the intact fibrous structure cannot be observed. The Schwann cells that provide nutrition and energy are not found around the nerve fibers. In addition, the PACAP27, MPAPO, and normal control groups had clear nuclear structure and uniform staining. It is obvious that the nucleus is round or oval, and the chromatin is evenly distributed. In the 0.9% NaCl group and the rb-bFGF group, the nucleus was dispersed, the staining was uneven, chromatin condensation was severe, and it is impossible to observe the outline of the nucleus clearly (Fig.2A).

We further investigated whether MPAPO can bind to optic nerve cells and other retinal cells at the tissue level to promote axonal regeneration. We added FITC-MPAPO to the injured cornea for 48 h, and the results showed that the green fluorescence (MPAPO) and red fluorescence (nerve fibers) were superimposed to form orange fluorescence (as indicated by the arrow in the figure), indicating that MPAPO can pass through the corneal stromal layer and the endothelial layer to reach the neuronal body and bind to the optic nerve cell. In addition to the nerve cells, a large amount of green fluorescence can be observed around other cells or on the surface. The results show that MPAPO can bind to it as long as there is a PAC1 receptor on the cell surface (Fig. 2B).

### MPAPO promotes corneal epithelial cell proliferation and injury repair

**Figure 3:**
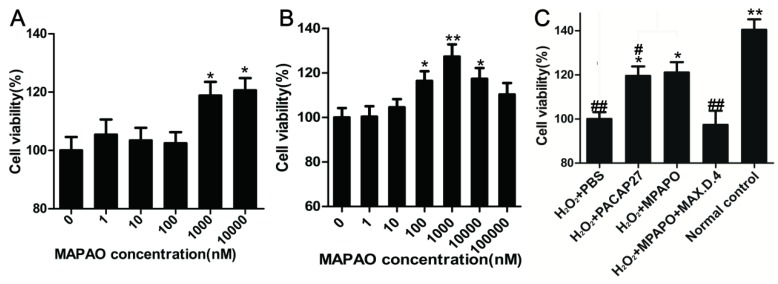
MPAPO promotes corneal epithelial cell proliferation and injury repair in mice. **(A)** The effects of different concentrations (0, 1, 10, 100, 1000 and 10000 nM) of MPAPO on the proliferation of normal corneal epithelial cells were investigated. **(B)** The repair effects of 0, 1, 10, 100, 1000 and 10000 nM MPAPO on injured corneal epithelial cells were studied. **(C)** Different experimental treatments promote corneal epithelial cell proliferation after injury (MAX.D.4 is a specific inhibitor of PAC1). *P<0.05, **P<0.01 compared to the 0 nM or PBS group; *^#^P*<0.05, *^##^P*<0.01 compared to the NC group. n=3.

Corneal epithelial cells were treated with different concentrations of MPAPO (0, 1, 10, 100, 1000, or 10000 nM) for 48 h, and cell viability was measured by the MTT assay. The data showed that low concentrations of MPAPO (below 100 nM) had no significant effect on the proliferation of corneal epithelial cells. When the concentration of MPAPO was higher than 1 μM, the cell proliferation rate reached approximately 20% (Fig.3A). The H_2_O_2_ injury corneal epithelial cell model was established by MTT assay. When the cells were injured with 400 μM H_2_O_2_ for 4 h, cell viability was maintained at approximately 50% (Supplementary Figure 2). In this case, a corneal epithelial cell injury model was established 36. The MTT assay was used to detect cell viability after treatment with different concentrations of MPAPO for 48 h. The experimental results show that when the injured cells were treated with 1 μM MPAPO, the cell proliferation effect was the most obvious, and the proliferation rate reached 20% or more. However, as the concentration of MPAPO increased further, the proliferation effect was slowly reduced. MPAPO had little effect on cell injury repair at a concentration of 100 μM (Fig.3B).

From the above results, the most suitable concentration of MPAPO to promote both corneal epithelial cell injury repair and cell proliferation is 1 μM. Subsequently, we investigated the repair effects of PACAP27 and MPAPO on injured corneal epithelial cells. The results showed that MPAPO and PACAP27 can significantly promote cell proliferation, and the cell proliferation rate reached approximately 20% compared with PBS. In the MAX.D.4 preincubation group (MAX.D.4 as a PAC1 receptor antagonist), the cell proliferation promoting effect disappeared compared with the PBS group (Fig.3C).

### MPAPO binds to the PAC1 receptor on corneal epithelial cells

**Figure 4:**
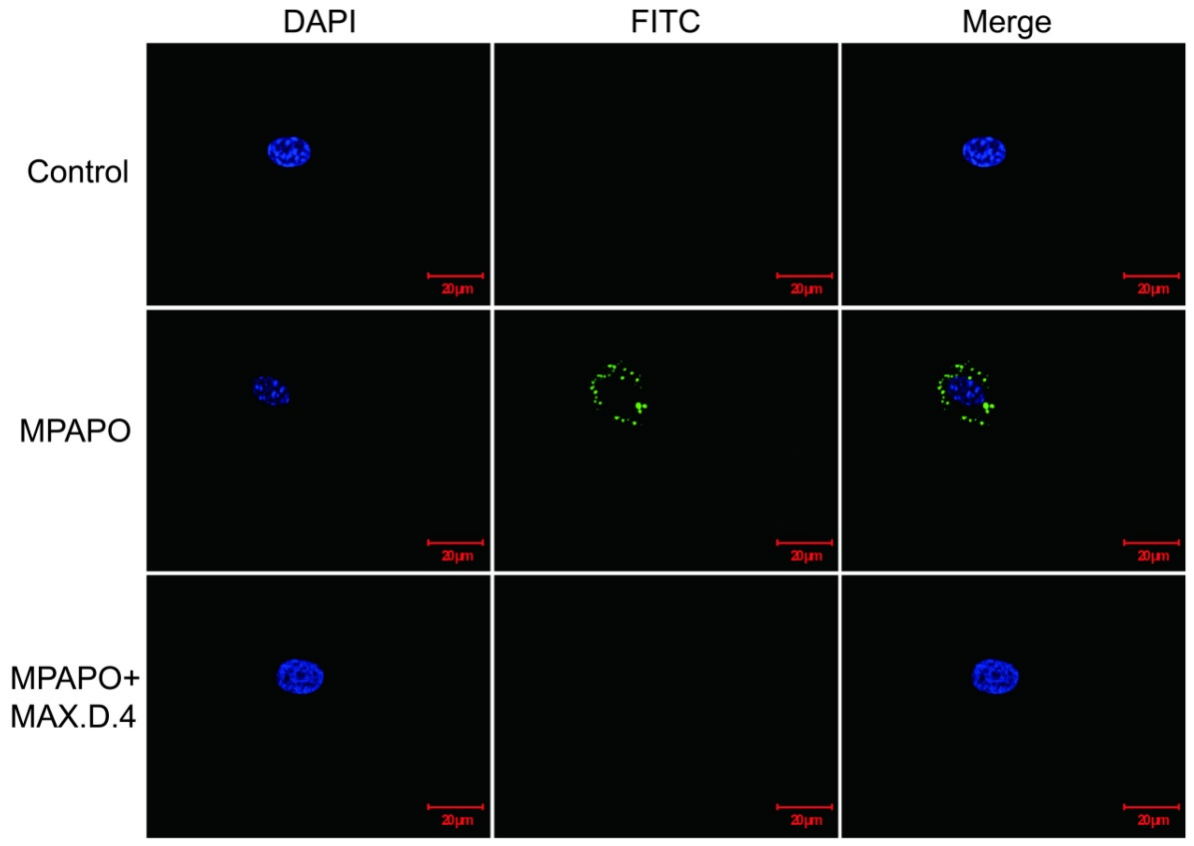
Immunofluorescence staining confirmed that MPAPO specifically binds to the PAC1 receptor on the cell surface. DAPI stained cell nuclei with blue fluorescence and FITC stained with green fluorescence, and merge is the result of the colocalization of the two kinds of fluorescence.

From the above experimental results, it was found that MPAPO can play a significant role in the repair of corneal wounds, but whether the modified MPAPO can bind to PAC1 is still a question. Whether MPAPO can bind to PAC1 on the cell surface was explored by immunofluorescence. The results showed that compared with the control group, FITC-MPAPO was able to bind to PAC1 on the cell surface and surround the cell membrane. With the addition of the PAC1 competitive inhibitor MAX.D.4, FITC-MPAPO could not effectively bind to the PAC1 receptor, which indicates that MPAPO can bind to the PAC1 receptor on the cell membrane efficiently and specifically, cannot bind to VPAC1 or VPAC2 (Fig.4).

### MPAPO promotes cell proliferation by regulating cell cycle progression

**Figure 5:**
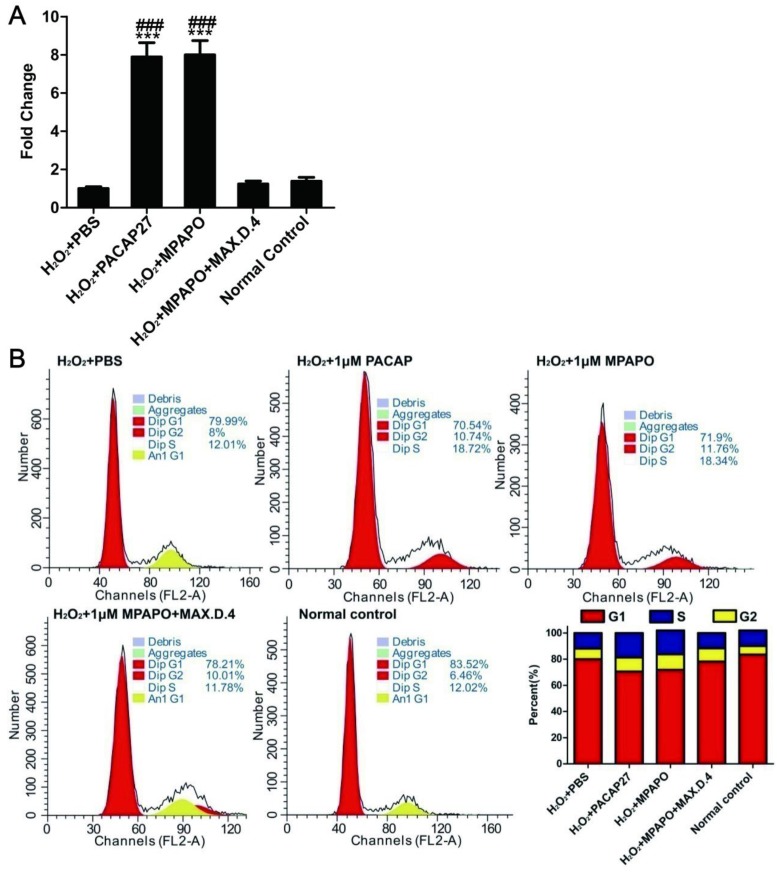
MPAPO regulates cell cycle progression in injured corneal epithelial cells. **(A)** The expression of cyclin D1 was verified by QPCR. **(B)** MPAPO changed the distribution of the corneal epithelial cell cycle. ***P<0.001 compared to the PBS group; *^###^P*<0.001 compared to the NC group. n=3.

To investigate the relationship between corneal epithelial cell proliferation and the β-catenin signaling pathway, we verified the expression of the downstream target genes of β-catenin by real-time PCR. The data showed that cyclin D1 was highly expressed in both the PACAP27 and MPAPO groups, which was approximately 8-9-fold higher than that in the PBS group, but the difference was not obvious in the other groups (Fig. 5A). Based on these results, it is speculated that MPAPO-repaired wound cells may be related to cell cycle progression. We examined the effect of MPAPO on the corneal epithelial cell cycle by PI single-staining flow. Flow cytometry analysis showed that the PACAP27 and MPAPO groups significantly changed the distribution of the cell cycle compared with the PBS group. In the PACAP27 and MPAPO groups, the G0/G1 phase decreased from 80% to 70%, while the S phase increased from 12% to 18% and the G2/M phase increased by 2%. The total cell cycle distribution of the MPAPO+MAX.D.4 group was not significantly different from that of the PBS group. Increases in the S phase and G2/M phase indicate that PACAP27 and MPAPO can alter the cell cycle distribution, thereby promoting cell proliferation (Fig. 5B).

### MPAPO repairs wounded corneal epithelial cells via the β-catenin signaling pathway

**Figure 6:**
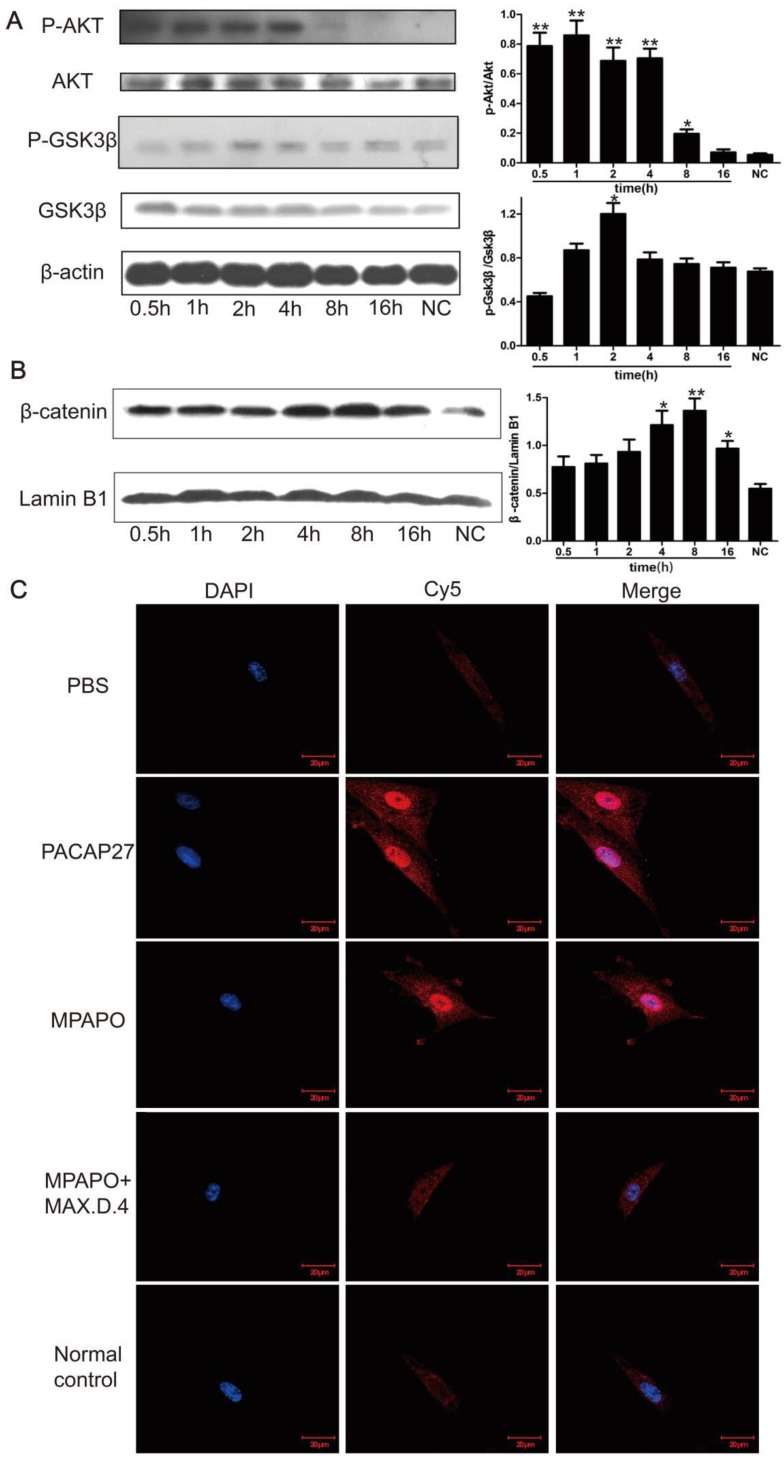
MPAPO is effective in activating the phosphorylation of AKT and GSK3β in corneal epithelial cells and promoting the entry of β-catenin into the nucleus. **(A)** Western blotting confirmed that MPAPO can promote the phosphorylation of AKT and GSK3β proteins. **(B)** Western blotting was used to study the nuclear importation of β-catenin, and Lamin B1 was used as the nuclear protein internal reference. **(C)** MPAPO promotes the entry of β-catenin into the nucleus of corneal epithelial cells after injury. DAPI is a nuclear dye with blue fluorescence. AF594 is a red fluorescent secondary murine antibody, and the primary antibody used is β-catenin. Merge is the result of the colocalization of the two types of fluorescence. *P<0.05, **P<0.01 compared to the normal control (NC) group. n=3.

To further investigate the relationship between MPAPO repair of the injured corneal epithelial cells and the β-catenin signaling pathway, we extracted the cell total protein at 0.5 h, 1 h, 2 h, 4 h, 8 h and 16 h after administration of MPAPO, and investigated the phosphorylation of AKT and GSK3β by Western blotting. The data showed that the phosphorylation rate of AKT after administration was very fast compared with the normal control group, reaching a high level within 0.5 h and continuing until 4 h. Phosphorylated AKT did not return to the same level as the normal control group until 16 h after administration. Phosphorylation of GSK3β was similar but not identical to AKT. During the period from 0 h to 2 h, the phosphorylation level of GSK3β gradually increased and remained at a relatively high level during the period from 2 h to 4 h. The phosphorylation level began to decrease until 8 h and returned to the normal control level after 16 h (Fig. 6A). The entry of β-catenin into the nucleus is considered to be a sign of activation of the entire signaling pathway. Western blotting was performed to verify the entry of β-catenin into the nucleus. The results showed that the increase of β-catenin into the nucleus was not obvious within 2 h after administration, but β-catenin levels in the nucleus began to increase significantly after 4 h and reached its maximum after 8 h. This protein gradually decreased in the nucleus until 16 h (Fig. 6B).

We treated the injured cells with PACAP27, MPAPO, MPAPO + MAX.D.4 and PBS for 8 h for immunofluorescence experiments to further investigate the entry of β-catenin into the nucleus. The results showed that the blue and red fluorescence superimposed into purple in the PACAP27 and MPAPO groups, and the signal was very strong. The entry of β-catenin into the nucleus is very obvious. The PBS, MAPAO+MAX.D.4 and normal control groups showed almost no red fluorescence (Fig. 6C). The results were consistent with the results of the Western blotting experiments described above.

**Figure 7:**
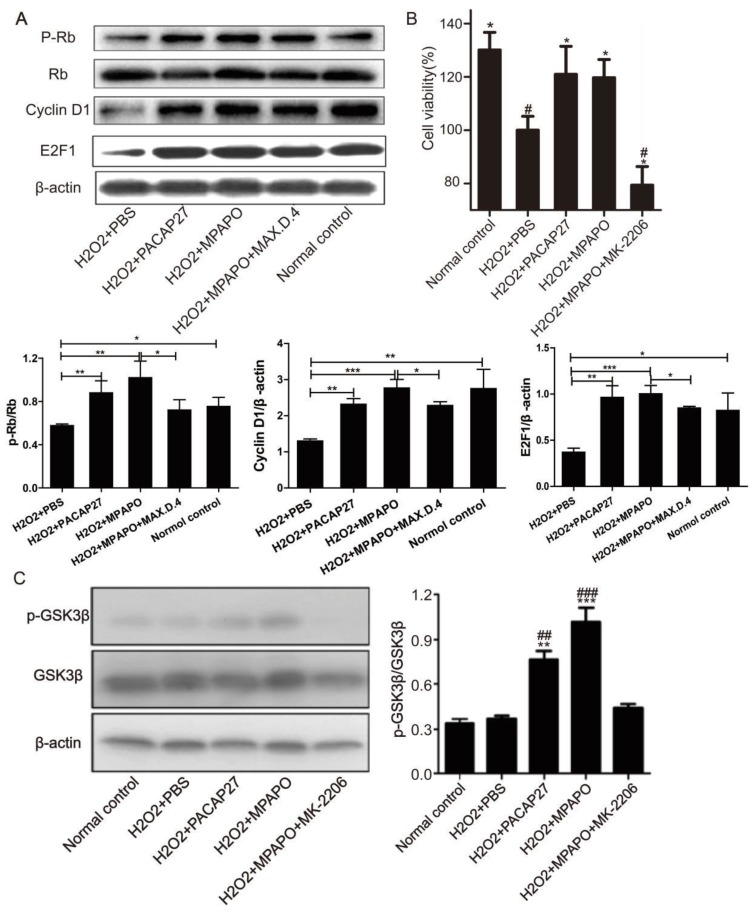
MPAPO promotes cyclin D1, E2F1 protein expression and Rb protein phosphorylation. **(A)** Western blotting verified the expression of the β-catenin signaling pathway related proteins. **(B)** MK-2206 inhibits the repair by MPAPO on injured corneal epithelial cells. **(C)** MK-2206 is able to inhibit the AKT phosphorylation of GSK3β. ^*^P<0.05, ^**^P<0.01,^ ***^P<0.001 compared with the PBS group. *^#^P*<0.05, *^##^P*<0.01, *^###^P*<0.001 compared to the NC group. n=3.

We then treated the injured cells with PACAP27, MPAPO, MPAPO+MAX.D.4 and PBS to investigate whether the downstream proteins of the β-catenin signaling pathway are differentially expressed. Western blotting results showed that compared with the H_2_O_2_ group, PACAP27 and MPAPO could significantly promote the expression of cyclinD1 and E2F1 and the phosphorylation of the protein Rb, and the MPAPO group has increased by approximately 2 times. While the MPAPO+ MAX.D.4 group had no obvious repair effect (Supplementary Figure 3). Moreover, the cyclin D1 expression level and the phosphorylation level of Rb in the MPAPO group were also higher than those in the PACAP27 group (Fig. 7A).

To determine whether phosphorylation-activated AKT can actually promote GSK3β phosphorylation, we used the AKT inhibitor MK-2206 for further research 37. The MTT results showed that when the concentration of MK-2206 reached 50 μM, the cell survival rate reached approximately 50% (Supplementary Figure 4). Then, we investigated whether AKT plays an important role in the entire signaling pathway. The MTT assay showed that the cell survival rate of the MPAPO + MK-2206 group significantly decreased compared with the MPAPO group, from 120% to 80%. The experimental results indicate that MK-2206 can inhibit the wound repair of MPAPO in corneal epithelial cells (Fig. 7B). Subsequently, we verified the phosphorylation level of GSK3β (GSK3β is a target protein of AKT) by Western blotting. The results showed that the phosphorylation level of GSK3β in the MPAPO + MK-2206 group was reduced by approximately halfway compared with the MPAPO group. GSK3β phosphorylation in the MPAPO+MK-2206 group was inhibited by MK-2266 (Fig. 7C).

### MPAPO promotes wound repair and axonal regeneration in trigeminal ganglion cells

**Figure 8:**
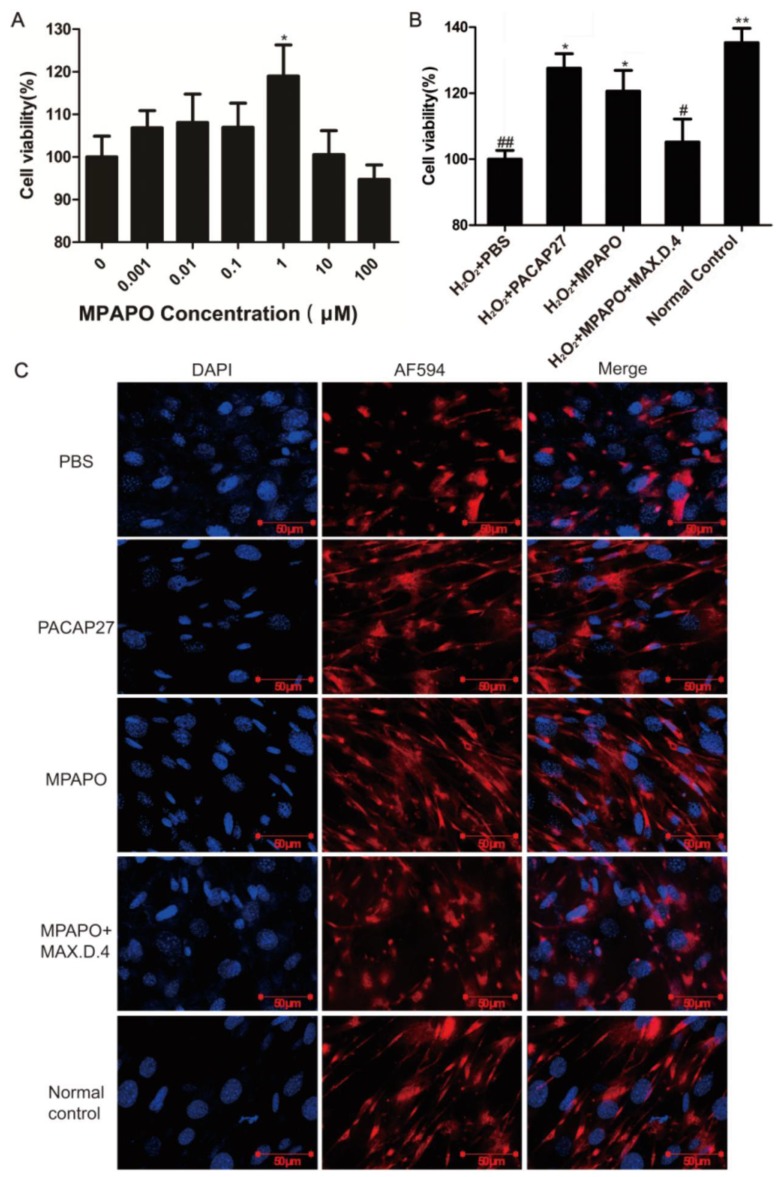
MPAPO is effective in promoting cell wound repair and axon regeneration. **(A)** The MTT assay explores the optimal concentration of MPAPO to promote trigeminal ganglion cell injury repair. ^*^P<0.05 compared to the 0 μM group. **(B)** The MTT assay was used to detect the injury repair effect of different administration methods on trigeminal ganglion cells. **(C)** Immunofluorescence staining was used to observe axonal regeneration in trigeminal ganglion cells. The nuclei were stained with blue fluorescent DAPI, and the trigeminal nerve fibers were stained with red fluorescent AF594. *P<0.05, **P<0.01 compared to the PBS group; *^#^P*<0.05,*^##^P*<0.01 compared to the normal control group. n=3.

We established an injury model of trigeminal ganglion cells to determine whether MPAPO can promote wound repair in trigeminal neurons. First, we established a trigeminal ganglion cell injury model with H_2_O_2_ by MTT assay. The results showed that 100 μM H_2_O_2_ injured trigeminal ganglion cells for 6 h, which is suitable for establishing a cell injury model (Supplementary Figure 5). Then, we set the MPAPO concentration gradient (0, 0.001, 0.01, 0.1, 1, 10, 100 μM) to treat the injured cells for 48 h. The results showed that when the concentration of MPAPO was less than 1 μM, the repair effect of the trigeminal ganglion cells became increasingly obvious with increasing concentration. When the concentration of MPAPO was 1 μM, the cell injury repair effect was the greatest, and the proliferation rate reached approximately 20% (Fig. 8A). Subsequently, we established five experimental groups to study whether MPAPO can promote trigeminal ganglion cell injury repair. As shown in the figure, the PACAP27 and MPAPO groups significantly promoted cell proliferation, and the cell proliferation rate was approximately 20%, while the MPAPO+MAX.D.4 group had no obvious repair effect (Fig. 8B). These results indicate that MPAPO can promote injury repair in trigeminal ganglion cells. Moreover, the injury repair effect can be inhibited by MAX.D.4.

It is unclear whether MPAPO can promote axonal regeneration in trigeminal ganglion cells. Immunofluorescence staining was used to visually observe the axon regeneration of trigeminal ganglion cells. The experimental results showed that the trigeminal ganglion cells of the PACAP27 and MPAPO groups had longer axons and were more densely distributed compared with the PBS group. The PACAP27, MPAPO and normal control groups showed elongated nerve fibers. Generally, each cell can grow two or three nerve fibers. The cells in the PBS group and the MAX.D.4 group clearly showed that the nerve fiber was not able to grow back after the injury, and there was no elongated nerve fiber (Fig. 8C). From the experimental results, it can be speculated that MPAPO and PACAP27 can promote the regeneration of axons in trigeminal ganglion cells.

### The mechanism of MPAPO promotion of axonal regeneration in trigeminal ganglion cells

**Figure 9:**
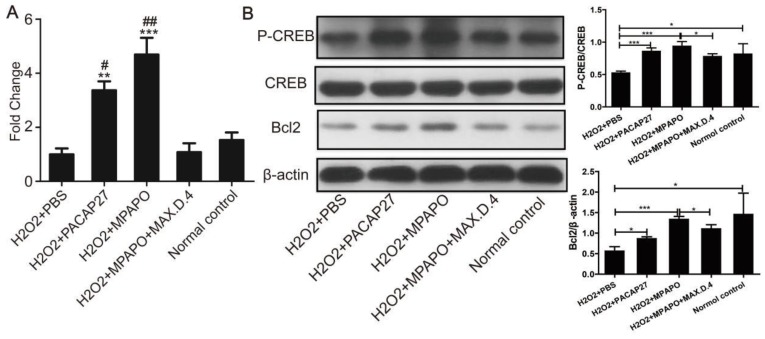
Study on the mechanism of MPAPO promoting injury repair of mouse trigeminal ganglion cells. **(A)** qPCR was used to verify the expression of Bcl_2_ in trigeminal ganglion cell axon regeneration. **(B)** Western blotting was used to verify that MPAPO promotes the proteins expression involved in trigeminal ganglion cell injury repair. *P<0.05, **P<0.01, ***P<0.001 compared to the PBS group; *^#^P*<0.05, *^##^P*<0.01 compared to the normal control group. n=3.

To study the mechanism of MPAPO promotion of axon regeneration in trigeminal ganglion cells, five experimental groups were established for RNA extraction, reverse transcription and qPCR to verify the expression level of Bcl_2_ after the establishment of the cell injury model. The data showed that the expression of Bcl_2_ in the PACAP27 and MPAPO groups was approximately 4 times higher than that in the PBS group. There was no significant change in the expression of Bcl_2_ in the MPAPO+MAX.D.4 and normal control groups (Fig. 9A). Western blotting verified the expression of Bcl_2_ and the phosphorylation of CREB after extracting the total cellular protein to further investigate the mechanism. The results showed that the phosphorylation level of the CREB protein in the MPAPO and PACAP27 groups was significantly increased (approximately 2-fold) compared with the PBS group. The expression of the Bcl_2_ protein in the MPAPO group was also approximately 2 times higher than that in the PBS group (Fig. 9B). In the MAX.D.4 group, there was almost no difference in the expression of the Bcl_2_ protein and the phosphorylation of the CREB protein compared with the PBS and normal control groups (Supplementary Figure 6). CREB acts as a phosphorylation substrate for PKA and Ser133 of CREB is a phosphorylation site of PKA, which thereby activates the transcriptional activity of CREB 38.

## Discussion

The cornea is the part of the eye that is exposed to the external environment and is therefore the most susceptible to damage from various insults. Corneal injury is one of the most common eye diseases, and if not treated properly, it could cause permanent injury and impaired vision. It is estimated that Americans have an eye injury rate of more than one million per year. These increasingly high numbers indicate a need for a better understanding of corneal healing mechanisms and the development of effective methods to accelerate and improve wound healing 8. Corneal injury is inevitably accompanied by injury to the corneal epithelium and the corneal nerve. In this sense, adequate wound healing after injury is critical to maintain the integrity of the corneal structure and function.

Recently, the main therapeutic strategies to promote corneal wound healing include the topical application of artificial tears, anti-inflammatory drugs, antibiotics, recombinant growth factors, cytokines and blood-derived products 39. Cytokines, such as basic fibroblast growth factor (bFGF), epidermal growth factor (EGF), keratinocyte growth factor, vascular endothelial growth factor (VEGF) and pigment epithelium-derived factor (PEDF), have been found to promote wound closure but are accompanied by the side effects of corneal angiogenesis 40-42. At home and abroad, the study of corneal wound healing is often performed in only one aspect, such as repairing only the corneal structure injury, and ignoring the regeneration of corneal nerves and blood vessels, or repairing only the corneal nerve, regardless of the corneal epithelium. These studies have shortcomings 43-45. Therefore, it is essential in corneal wound repair to promote both the healing of corneal wounds and the regeneration of corneal nerves.

PACAP and its receptors are widely distributed in the central nervous system and peripheral nervous system. PACAP plays an important role in nerve growth, development, differentiation, apoptosis and regeneration 46. In nerve cells, PACAP activates related signaling pathways through the PAC1 receptor, promotes neuronal survival, inhibits neuronal cell apoptosis, and protects neurons. PAC1 activates the cAMP and PKA signaling pathways to promote neuronal differentiation 25. Due to the short half-life of PACAP *in vivo* and the characteristics of its receptor binding site, our group designed a mutant polypeptide of PACAP27 in the early stage and named it MPAPO 24. Recently, the use of proteins and peptides as human therapeutic drugs has increased rapidly 47. Peptides are considered to be viable therapeutic drugs because of their higher specificity compared to large molecules and smaller size relative to antibodies 48. In addition, the peptide MPAPO shows better stability, a longer half-life, stronger specificity and fewer side effects than PACAP.

In recent years, many studies have shown that PACAP can promote the proliferation of neural stem cells 49, TtT/GF cells 50, corneal epithelial cells and trigeminal ganglion cells, and promote the repair of corneal epithelial damage 51, 52. We synthesized the PACAP27-derived peptide MPAPO and found that it can effectively promote corneal wound repair and tear secretion 24. In this study, we further explored the effects and mechanisms of MPAPO to promote corneal wound repair. It was found that MPAPO can promote the repair of corneal wounds in mice and does not promote the formation of blood vessels. There are no blood vessels in the cornea and blood vessels can affect light transmission in the eyes, thus affecting vision 53. Therefore, the side effects of MPAPO to promote corneal wound repair are relatively small. From the results of the immunofluorescence staining experiments, MPAPO can promote corneal nerve regeneration, which is of great significance for corneal wound healing and vision. In addition, MPAPO had a good effect on corneal epithelial cell proliferation and injury healing at a low concentration (1 μM); it also promotes wound healing and axonal regeneration in trigeminal ganglion cells, while the effects at high concentration are slowly reduced. The reason for this result may be that high concentrations of MPAPO have greater cytotoxicity and cell resistance than low concentrations. Therefore, a low concentration of MPAPO can promote corneal wound repair by promoting corneal epithelial cell proliferation and trigeminal nerve axon regeneration.

**Figure 10:**
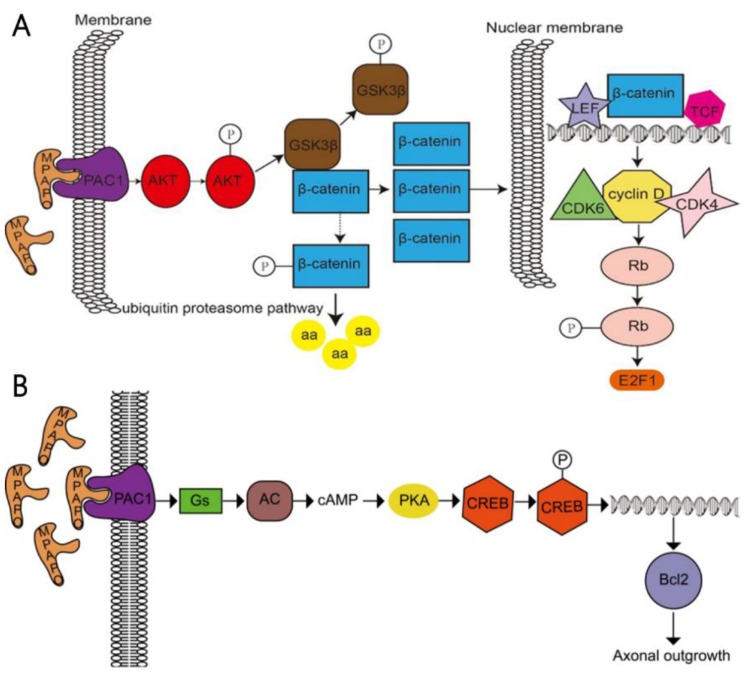
Study on the mechanism of the MPAPO promoting of corneal wound repair and axonal regeneration **(A)** MPAPO activates the classic Wnt/β-catenin signaling pathway through the PAC1 receptor. **(B)** Schematic diagram of the mechanism by which MPAPO promotes axonal regeneration in trigeminal neurons.

In a further study, it was found that MPAPO promotes the repair of corneal epithelial cell damage by MPAPO-mediated PAC1 receptor activation of the β-catenin signaling pathway (Fig. 10A). MPAPO specifically binds to the PAC1 receptor and activates the phosphoinositide and cAMP pathways to further phosphorylate and activate AKT. Activated AKT phosphorylates GSK3β, which once phosphorylated, it loses the ability to phosphorylate β-catenin. β-Catenin enters the nucleus, recruits cyclin D1, and CDK4/6 bind to the nucleus and phosphorylates Rb. Phosphorylated Rb activates E2F and other gene transcription factors, which initiates cellular DNA replication. Cells enter the S phase through the G1 phase, which in turn affects cell cycle progression and promotes cell proliferation. However, this study shows that MPAPO promotes trigeminal nerve axon regeneration. MPAPO binds to the PAC1 receptor and activates adenylate cyclase activity. Subsequently, cAMP activates protein kinase A activity and CREB phosphorylation. Finally, the phosphorylated CREB promotes the expression of Bcl_2_ and trigeminal ganglion cell axon regeneration (Fig. 10B). Other studies have shown that negative regulation by the PIP3/Akt/mTOR pathway can also promote neuronal survival and axonal regeneration 54. Nerve repair of the cornea is a dynamic process that is critical to the restoration of the overall structure and function of the cornea. The mechanism of nerve repair still needs more in-depth and extensive research to better clarify the healing response and related mechanisms after corneal injury, to find the best way to promote corneal nerve repair and obtain long-term and effective effects.

We have explored the role and mechanism of MPAPO in corneal wound repair *in vivo* and *in vitro* to gain a more comprehensive and in-depth understanding of the benefits of MPAPO. We found that, by binding to the PAC1 receptor, MPAPO activates the β-catenin signaling pathway to promote corneal wound repair and promote corneal nerve axon regeneration through the cAMP/PKA signal pathway. MPAPO is expected to provide a new direction for the treatment of corneal diseases and lay a solid foundation for future clinical applications.

## Supplementary Material

Supplementary figures and tables.

## Abbreviations

PACAP: Pituitary adenylate cyclase-activating polypeptide
VIP: Vasoactive peptide
NGF: Nerve growth factor
GDNF: Glial cell-derived neurotrophic factor
PCP: Planar cell polarity
GSK3β: Glycogen synthase kinase 3β
PLC: phospholipase Cβ
IP3: inositol 1,4,5-trisphosphate
PKA: protein kinase A
CK1: Casein Kinase 1
β-TrCP: β-transducin repeats-containing proteins
LRP: Lipoprotein receptor-related protein
TCF: T-cell factor
LEF: Lymphoid enhancing factor
EDTA: Ethylenediaminetetraacetic acid
bFGF: Basic fibroblast growth factor
DMSO: Dimethyl sulfoxide
DMEM: Dulbecco's Modified Eagle Medium
MTT: 3- (4,5-cimethylthiazol-2-yl)-2,5-diphenyl tetrazolium bromide
DEPC: Diethy pyrocarbonate
DAPI: 4',6-diamidino-2-phenylindole
FITC: Fluorescein isothyocyanate
PKA: protein kinase A
EGF: Epidermal growth factor
VEGF: Vascular endothelial growth factor
PEDF: Pigment epithelium-derived factor
