# Supplementary material for: The PACAP-derived peptide MPAPO facilitates corneal wound healing by promoting corneal epithelial cell proliferation and trigeminal ganglion cell axon regeneration
Source: Int J Biol Sci. 2019 Oct 15;15(12):2676–91. doi: 10.7150/ijbs.35630 (PMC6854382; doi:10.7150/ijbs.35630)
Supplement: Supplementary file 1 — Supplementary figures and tables. [file ijbsv15p2676s1.pdf]

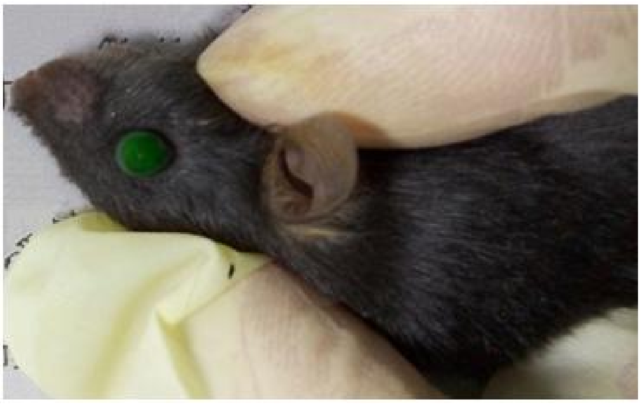

Supplementary Figure 1. Mouse cornea after injury. After sodium fluorescein staining, it was washed with physiological saline. A round green area formed in the middle of the mouse eyeball was the cornea of the injured mouse eye, and uniform staining was observed.

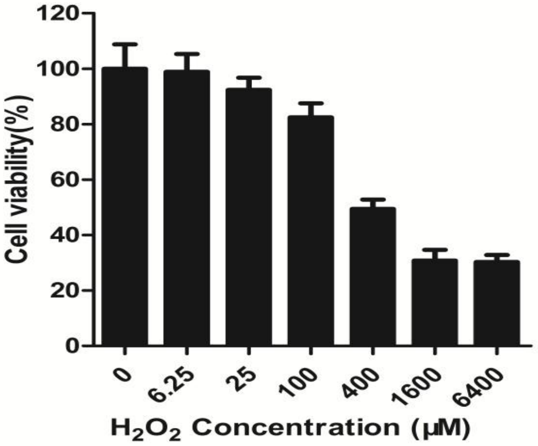

Supplementary Figure 2. A corneal epithelial cell injury model was established by treating normal cells with different concentrations (0, 6.25, 25, 100, 400, 1600, and 6400 μM) of H_2O_2. The effect of cell injury in low concentration (below 25μM) was not obvious. With the increase of H_2O_2 concentration, the cell viability decreased sharply, the survival rate was 80% at 100μM, and the survival rate was 50% at 400μM. When the concentration of H_2O_2 was further increased, the cell viability decreased further. The survival rate of cells at 1.6 mM and 6.4 mM was only about 20%.

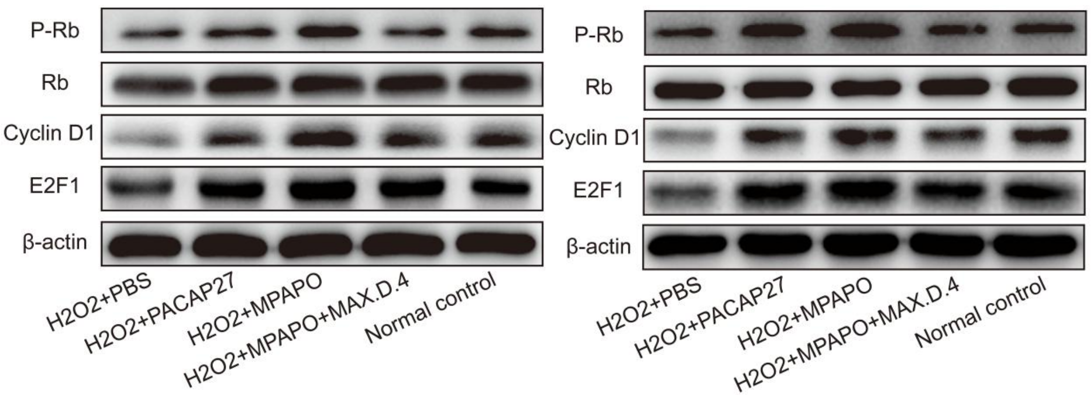

Supplementary Figure 3. Western blotting verified the cyclin D1, E2F1 protein expression and Rb protein phosphorylation..

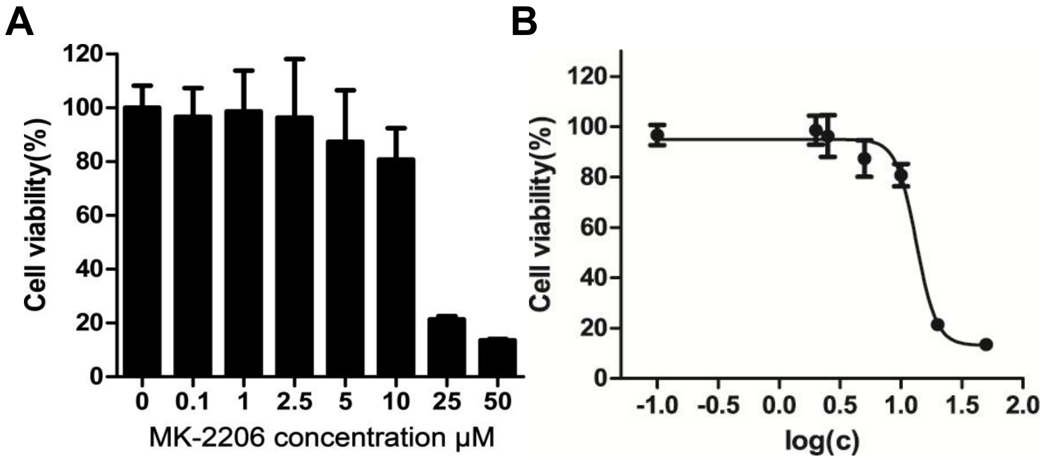

Supplementary Figure 4. MTT assay for the lethal dose of MK-2206 on mouse corneal epithelial cells. (A) When the concentration of MK-2206 reached 50 µM, the cells all died. The cell survival rate from 10µM to 25µM decreased from 80% to 20%. (B) According to the results of MTT experiment, the cell survival rate is the ordinate, the logarithm of the base 10 is lg(c) as the abscissa, and the IC50 calculated by curve fitting is 13.8 µM.

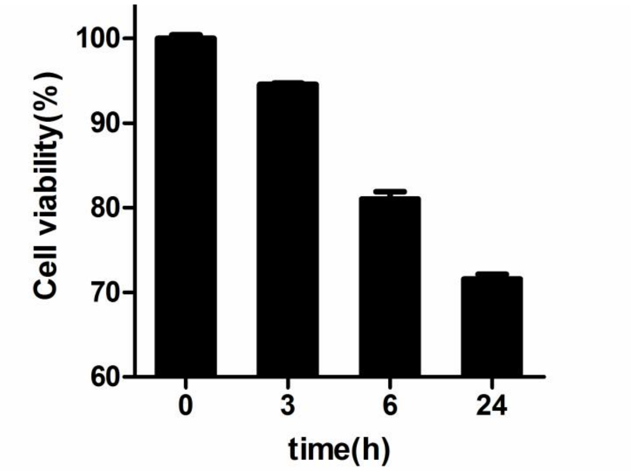

Supplementary Figure 5. MTT assay was used to study the survival rate of cells treated with H_2O_2 at 0h, 3h, 6h and 24h. The data showed that the cell survival rate gradually decreased with time after administration, the cell survival rate reached 80% at 6h, and 70% at 24h.

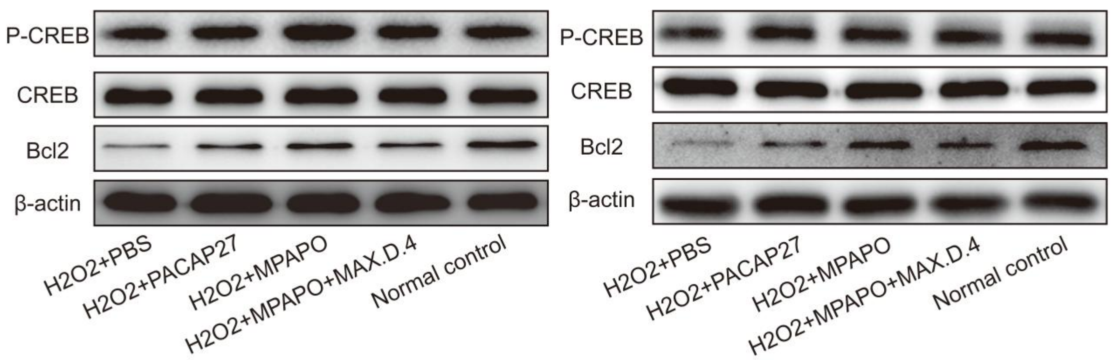

Supplementary Figure 6. Western blotting was used to verify that MPAPO promotes the CREB and Bcl2 proteins expression involved in trigeminal ganglion cell injury repair.
